# Thin-Layer TiO_2_ Membrane Fabrication by Condensed Layer Deposition

**DOI:** 10.3390/ma17174436

**Published:** 2024-09-09

**Authors:** Mohammed M. Numaan, Ahmed M. Jasim, Yangchuan Xing, Maria M. Fidalgo

**Affiliations:** 1 Department of Civil and Environmental Engineering, University of Columbia, Columbia, MO 65211, USA; mohammed.m.numaaan@tu.iq; 2 Department of Chemical and Biomedical Engineering, University of Missouri, Columbia, MO 65211, USA; amjtg6@missouri.edu (A.M.J.); xingy@missouri.edu (Y.X.)

**Keywords:** ceramic membranes, ultrafiltration, coatings

## Abstract

A novel approach to the fabrication of thin-film supported metal oxide membranes was investigated. Nanocoatings were obtained by the condensed layer deposition of TiO_2_ on tubular microporous supports, applying multiple consecutive layers of TiO_2_/polyaniline. The surface, cross-sectional structure, and morphology of the materials were investigated by electron microscopy. Their membrane-related properties were explored by permeability measurements, rejection, and fouling analysis, using polyethylene glycol (PEG) as test molecules. The SEM images showed that TiO_2_ was successfully deposited on the surface, creating a layer with partial coverage of the support after each layer was deposited; consequently, the permeability of the membranes decreased gradually. Overall, the results of the flux and permeability of the membranes confirmed the coating. The transmembrane pressure (TMP) increased with each coating layer, while the rejection of the membrane showed gradual improvement.

## 1. Introduction

Separation processes are key to the feasibility of water treatment and resource conservation strategies. Emerging contaminants and impacted water sources pose additional challenges to the provision of safe drinking water to a growing population. More sustainable systems call for circular economies, while valuable materials are recovered from complex waste streams. The membrane process can provide answers to these needs, and, in particular, ceramic membranes are ideal candidates for advanced separation processes due to their relatively high thermal and chemical stability, potential catalytic activity, erosion resistance, and non-compactability under high pressure [1]. 

Ceramic membranes are generally composed of a porous ceramic material that acts as support and provides mechanical strength, and a thin separation layer that provides functionality. An ideal support material is relatively low-cost, uniform, inert, and highly permeable. Alumina microfiltration membranes are commonly used as support materials for ceramic ultrafiltration and nanofiltration membrane fabrication due to their availability and cost. Although ceramic membranes may present higher initial costs than their polymeric counterparts, an appropriate comparison should not only consider membrane area but also durability, mainly with respect to sustained separation performance and fouling [2,3]. Generally, membrane fouling occurs in forms of foulant adhesion/deposition [4] and the formation of a gel/cake layer [5]. As a result, foulants accumulate and block the pores, leading to a reduced permeate flow, decreasing the life of the membrane and increasing the overall cost [6]. Strategies to manage fouling include surface modification, chemical cleaning, and pH variations [7,8]. The selection of a successful approach for fouling mitigation depends on the physicochemical characteristics of the membrane surface and feed solution. 

Inorganic–organic nanocomposite membranes have been attracting interest in the field of sustainable water purification by using different techniques, such as electrospinning [9], layer-by-layer assembly [10], physical coating [11], and self-assembly [12]. Consequently, incorporating nanoparticles consisting of metal oxides such as Al_2_O_3_, Fe_2_O_3_, SiO_2_, and TiO_2_ [13] in polymer matrices will enhance the physicochemical properties and functionalities of membranes [14,15]. However, care should be taken in selecting combinations that add advantages while avoiding too many of the disadvantages of the polymeric materials. For example, photocatalytical membranes have been proposed based on TiO_2_ nanoparticles added to a polymeric matrix, but the produced reactive oxidant as well as the UV radiation during operation are harmful to the polymeric material, reducing the life of the membrane. 

Supported inorganic thin separation layers have been obtained in the fabrication of ultrafiltration and nanofiltration ceramic membranes. Very thin active layers are desirable to minimize hydraulic resistance and obtain high fluxes, preferably below 1 µm. Available technologies to achieve this goal include sol–gel coating and chemical vapor deposition (CVD) processes. The cost and the ease of scale-up to large surfaces and more complex support structures (tubular vs. flat) are hurdles for these technologies, as well as achieving precise control over the thickness and conformation of the layer [1]. Recently, a novel condensed layer deposition (CLD) process for the manufacture of nanoscale coatings on solid substrates has been reported [16]. The CLD process is a promising technology that can potentially overcome current limitations in the fabrication of membrane thin active layers. Substrates are immersed in an oil phase to which water is added and, as it reaches saturation, forms a nanoscale water film on the solid. Then, a chemical precursor is injected and reacts with the water to form metal oxide coatings. 

The objective of this work was to fabricate asymmetric ceramic membranes in the ultrafiltration range using the CLD process and to assess their application in water treatment. Herein, we report on the fabrication of thin-film supported metal oxide membranes by CLD of TiO_2_ nanostructured layers. Tubular microporous ceramic supports were coated with multiple consecutive layers of TiO_2_/polyaniline. The surface and cross-sectional structure and morphology of the materials were investigated by electron microscopy. Their properties as membrane filters were explored by permeability, separation, and fouling determinations, using polyethylene glycol (PEG) as the test molecule. 

## 2. Materials and Methods

### 2.1. Materials

All reagents were analytical grade and used as purchased without additional purification. Polyethylene glycol (PEG) C_2n_H_4n+2_O_n+1_ (MW 20,000 and 200,000 Da), potassium hydrogen phthalate (≥99.95%), ammonium persulfate (≥98%), and titanium isopropoxide (99.9%) were purchased from Sigma-Aldrich (St. Louis, MO, USA). Hydrochloric acid (38% analytical grade) and heptane (≥99%) were acquired from Alfa Aesar (Tewksbury, MA, USA). Aniline liquid (≥99%) was purchased from Fisher Scientific (Pittsburgh, PA, USA). Ultrapure water (18.2 MΩ·cm) was used in all the experiments and provided by the Thermo Scientific Barnstead^TM^ E-pure^TM^ ultrapure Water Purification System (Waltham, MA, USA). Ultrapure water was used in all experiments from Type I (resistivity 18 MΩ·cm) and obtained from a Barnstead E-pure ultrapure Water Purification System (Thermo Scientific, Waltham, MA, USA).

### 2.2. Membrane Fabrication

Macroporous alumina ceramic tubes (length 4 cm, OD 9.5 mm, and ID 3.2 mm) used as membrane supports were kindly provided by Dr. Guillermina Gentile, Instituto Tecnológico de Buenos Aires. The supports were subjected to 1, 2, 3, or 4 nanolayer deposition cycles of TiO_2_, following a condensed layer deposition process [16,17]. The tubular supports were placed in 200 mL of heptane under gentle stirring for 10 min. Then, 0.5 mL of 0.5 M of titanium isopropoxide was added and left for 30 min under an ultrapure N_2_ flow to prevent any loss of the precursor due to air moisture. Polyaniline (PANI) was introduced as a second coating agent between layers to enhance the bonding between the TiO_2_ coatings. In a typical approach, 500 μL of aniline solution was first dissolved in 2 mL of the 0.5 M HCl solution, and then the TiO_2_-coated supports were added under stirring for 30 min. After the above mixture was confirmed to be homogenous, 5 mL of the 0.5 M ammonium persulfate (as an oxidizer) solution was added dropwise with vigorous stirring. The suspension was stirred overnight, filtered, washed with DI water, and finally dried at 80 °C overnight. The process of TiO_2_/PANI coating was repeated to obtain the multilayer membranes.

### 2.3. Characterization

#### 2.3.1. Material Characterization 

Scanning electron microscopy (SEM) (Quanta 650 FEG, FEI, Hillsboro, OR, USA) was used to analyze the visual information of the surface and cross-sectional morphologies of the coated tubular membranes. Multiple images were observed in different regions of the samples to ensure the images were representative of the material features. ImageJ [18] software (version 1.52n) was used to determine the surface pore sizes for the support and fabricated membranes.

#### 2.3.2. Membrane Permeability

In the determination of clean water flux (J_w_), ultrapure water was filtered through the membrane at a constant pressure maintained by a water column, with discharge at atmospheric pressure and room temperature (25 °C). Experiments were conducted in triplicates. 

The permeate was collected in a graduated cylinder and the volume was recorded vs. time. The water flux (J_w_) was calculated by applying Equation (1):

J_w_ = V/(A·t) (1)

where J_w_ is the water flux; V is the permeated water volume; A is the effective filtration area of the membrane (0.01 m^2^); and t is time. The flux was determined from the slope of the V(t) line and the membrane permeability (P_m_) was calculated by Equation (2):

P_m_ = J_w_/P (2)

where P is the applied pressure.

#### 2.3.3. Membrane Filtration Experiments

**Figure 1:**
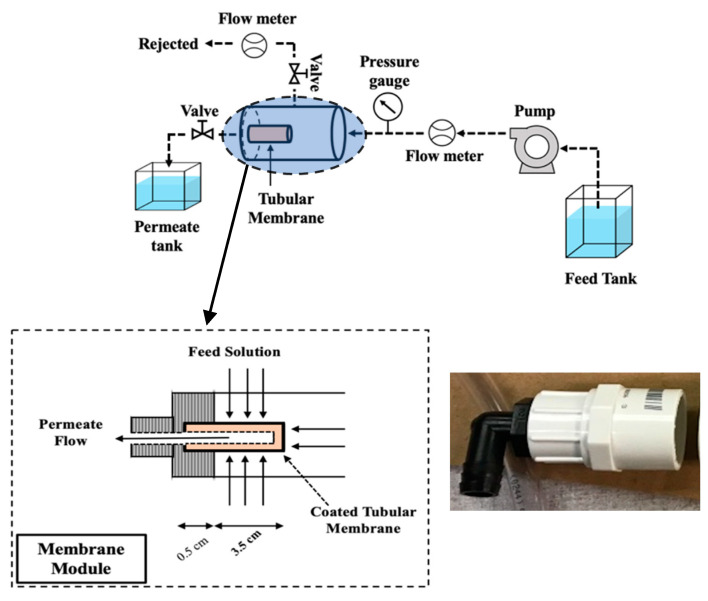
Schematic of the experimental setup and the membrane module.

Membrane filtration tests were conducted in cross-flow mode. The experimental setup is shown in Figure 1. A variable-speed peristaltic pump was used for the feed; permeate and feed flows were controlled and pressure at the module was monitored (Cole-Parmer, Vernon Hill, IL, USA). A custom-made membrane module was used to house the ceramic membrane (see inset in Figure 1). 

The feed solution was pumped into the system at a constant inflow rate of 33 mL/min and a retentate flow rate of 22.5 mL/min. The operating flow rates were manually adjusted using the flow control valves installed in the flowmeters and the valve in the permeate flow line to assure constant values. The permeate samples were collected periodically through the experiments.

The rejection and fouling propensity of the membranes were investigated using test aqueous solutions of PEG molecules with different molecular weights: 20,000 Da and 200,000 Da. An initial concentration of 25 ppm was selected in order to promote accumulation and fouling on the surface of the membranes. Experiments were run until a significant increase in the transmembrane pressure was observed due to fouling, or the pressure required to maintain constant flow was higher than allowable. The permeate was sampled periodically throughout the experiments and the concentration of PEG was measured. Samples were taken from the first 100 mL of permeate obtained in each experiment. The rejection percentage for each test molecule (PEG_20_, PEG_200_) was calculated using Equation (3):

R_PEGi_ (%) = (1 − C*_f_*/C*_i_*)·100 (3)

where *C_f_* and *C_i_* are the concentration of PEG in the permeate and feed flows, respectively.

PEG concentrations were determined from total organic carbon analysis using a PC-Controlled Total Organic Carbon Analyzer (Shimadzu Corporation Analytical & Measuring Instruments Division, Kyoto, Japan). Calibration curves (concentration vs. TOC) of the PEG solutions were established in order to convert the obtained value to actual PEG concentrations.

Fouling was investigated by monitoring the evolution of transmembrane pressure while operating at a constant feed flow of 33 mL/L and a retentate flow of 22.5 mL/L. All experiments were conducted at room temperature (25 °C). After the filtration stage, the membranes were subjected to physical cleaning by backwashing with ultrapure water (18.2 MΩ·cm) and the reevaluation of the clean water flux. Experiments were conducted in triplicates.

## 3. Results

### 3.1. Surface Morphology

**Figure 2:**
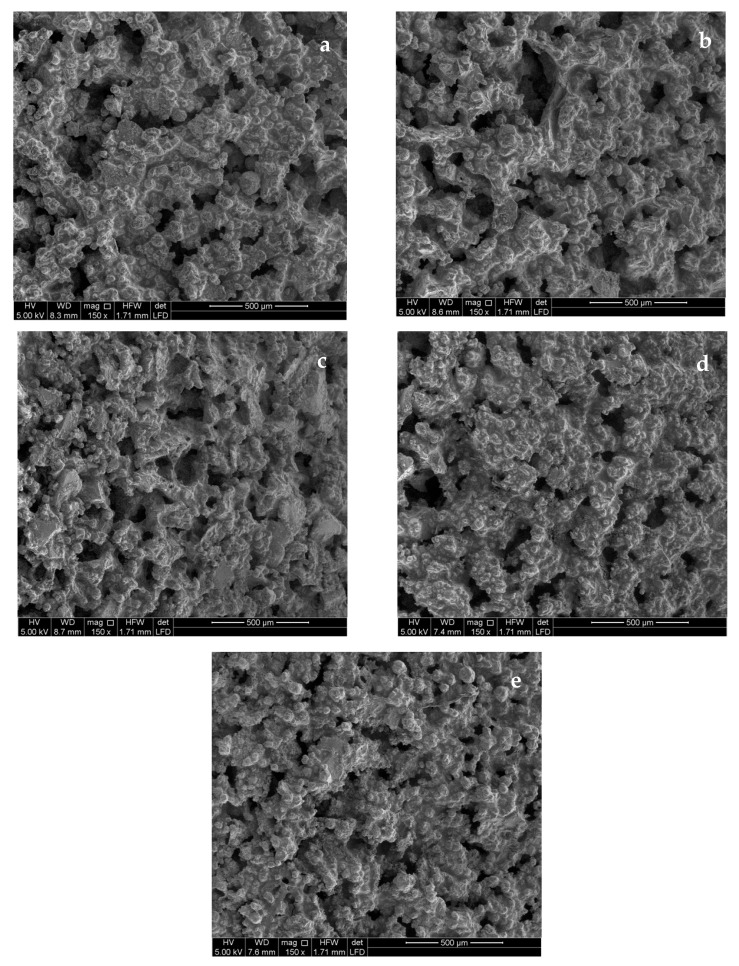
SEM images of uncoated membrane surfaces (**a**) and surfaces coated with (**b**) 1 TiO_2_ layer, (**c**) 2 TiO_2_ layers, (**d**) 3 TiO_2_ layers, and (**e**) 4 TiO_2_ layers.

SEM images of the surface of the tubular supports showed a microporous structure with visible grains in the micron range. Figure 2a–e show the SEM images of the surface of the coated and uncoated tubular membranes. The images show similar morphology, dominated by the larger grain size of the supports, although smaller-scale features appear on the surface progressively as the number of TiO_2_ nanocoatings increases. 

In order to quantify these observations, we applied a computer-assisted image analysis to the measurement of surface openings as an indirect measurement of the degree of coverage of the coating layer. The Image J analysis confirmed the observations. The analysis showed that the surface pore diameters of the uncoated tubular membrane ranged between 117 μm and 331 μm, and they decreased gradually, measuring in the range of 92.26–226.34 μm, 74.5–166.8 μm, 41.31–103.26 μm, and 19.95–67.43 μm for one-, two-, three-, and four-layer coatings, respectively. 

The decrease in the apparent surface pores was due to the nanoscale deposition of TiO_2_/polyaniline layers partially covering the surface topology. The progressive change in the pore range size indicated that although TiO_2_ was being formed on the alumina grains, there was incomplete coverage of the surface as its surface pore structure was being continuously modified by subsequent coatings. 

**Figure 3:**
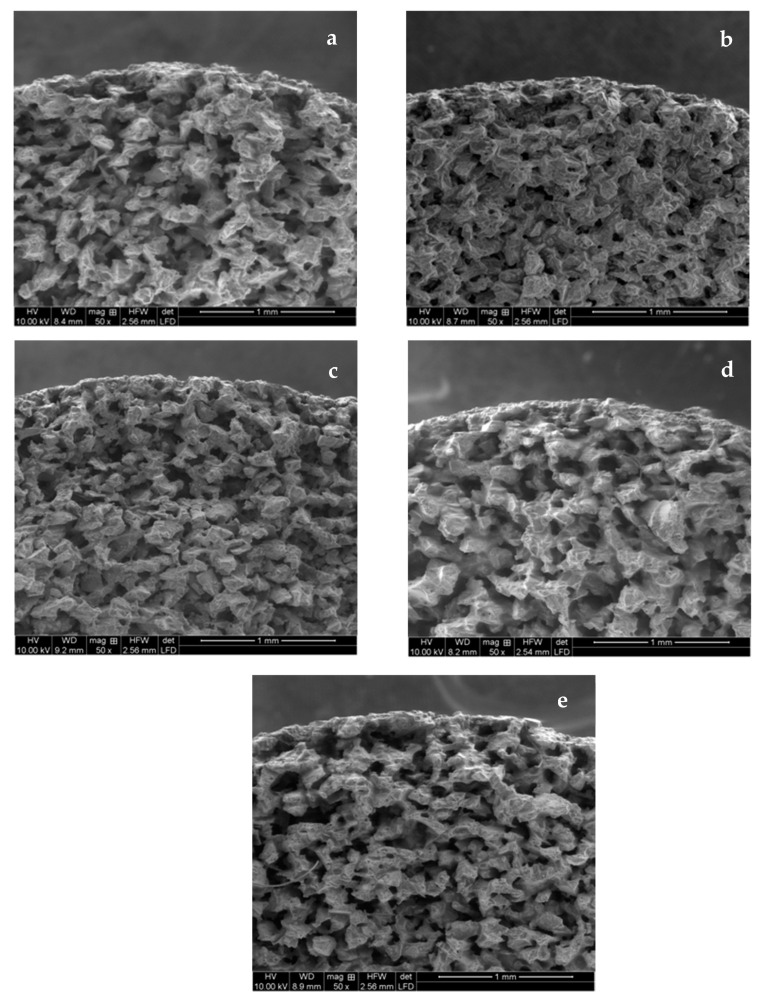
SEM images of membrane cross-sections of (**a**) uncoated support and support coated with (**b**) 1 TiO_2_ layer, (**c**) 2 TiO_2_ layers, (**d**) 3 TiO_2_ layers, and (**e**) 4 TiO_2_ layers.

Figure 3a–e present the cross-sectional images of all the membranes fabricated in this study. The thickness of the CDL can be controlled by the thickness of the condensed water layer if enough precursor is added in a complete reaction; assuming a similar surface area of the support materials, the thickness of each layer is expected to remain constant and conform to the outer surface of the substrate [16].

The SEM images showed an abundance of microporosity, but the deposited TiO_2_ layers are not discernible, as can be expected given the macroscale porosity and surface roughness of the support and the nanoscale of the condensed layer deposited. The images are compatible with the expected nanoscale thickness of the coating layers, without any indication of areas with accumulation or protrusions. 

Overall, the SEM images provided indication that nanoporous structures were created successfully on the support surface and that they gradually progressed with an increasing number of coating layers.

### 3.2. Permeability Measurements

**Figure 4:**
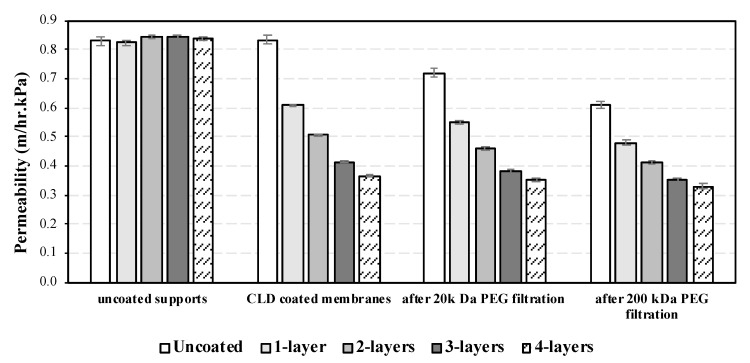
Water permeability of support and coated membranes with 1, 2, 3, and 4 layers; CLD coated clean membranes; after PEG filtration and hydraulic cleaning.

Water permeability was measured for the clean membranes before and after coating them with TiO_2_ and TiO_2_/PANI, and after being applied to the filtration of PEG solutions and subjected to hydraulic cleaning of the surface. The results are shown in Figure 4 as averages of three runs. 

Membrane permeability declined continuously with the increasing number of TiO_2_ layers applied. As expected, successive coating treatments produced a decrease in the permeability as TiO_2_ particles accumulated, leading to narrowing surface pore entrances. The largest drop was observed after the first coating, while more modest permeability losses were detected after subsequent coatings. This observation allows us to infer that the active TiO_2_ layer gradually formed on the surface of the support; although this layer resulted in increased hydraulic resistance for the asymmetric membrane, the reduction in pore sizes offers additional functionality for the material as a filter in the ultrafiltration/nanofiltration range. 

The potential changes in membrane permeability during normal operation was studied by conducting a filtration stage followed by hydraulic cleaning. The membranes were applied to the filtration of PEG solutions (MW 20,000 Da and 200,000 Da) and were flushed with ultrapure water immediately after use. As expected, a slight decrease in the permeability was observed, which can be attributed to irreversible fouling fraction or foulant accumulation inside the pore structure that may have been inaccessible to the hydraulic cleaning. 

The highest recovery of flux and permeability was observed for the four-layer membranes, with 96% and 90% of the initial permeability for 20 kDa and 200 kDa PEG solutions, respectively. The relative permeabilities under clean and used conditions are summarized in Table 1 for all fabricated materials. 

The diminishing effect on permeability becomes less pronounced with each nanolayer deposited, suggesting that while particles may have blocked or reduced a fraction of the surface porosity, there was a fraction of the pores that remained unaffected. It is possible to hypothesize that the nanoscale nature of the deposited particles favors relatively smoother sections of the surface and fails to reach deep inside the valleys in the support surface and, therefore, are unable to grow an integral layer of material on a rough ceramic surface such as the one used in this study. 

The PEG removal showed a similar effect in all coated materials, with an 80–96% permeability loss after the membrane was placed in operation/washed. The relationship between the number of coatings and the degree of irreversible fouling, expressed as the percentage of clean membrane permeability not recovered during hydraulic cleaning, is consistent with the absence of continuous pore reduction, the formation of a patchy nanoscale TiO_2_ coverage growing in size and depth, and with some areas of the support surface remaining unmodified by the condensed layer deposition. Transmembrane pressure increased sharply during filtration; however, after backwashing with ultrapure water, the pressure decreased to values comparable to the initial ones. 

The surface of coated membranes is expected to include a variety of functional groups, such as C=C, C=N, C–N^+^, and N–H [19]. Therefore, the PEG molecules in the feed solution may become adsorbed onto the membrane surface by forming hydrogen bonds between the surface OH^−^ groups on the metal oxide (Al_2_O_3_, TiO_2_ and/or TiO_2_/PANI, and the lone pair of electrons in ether O in PEG [20,21]). For these bonds to form, the surface OH- groups should exhibit acidic behavior, as determined by the electron distribution in the metal–O bond; the more electron density displaced to the O atom, the stronger the attraction for the proton, and the less acidic the group [22]. Al atoms have higher electronegativity than Ti, and therefore, the Al-O bond is expected to be more covalent in nature than Ti-O and show a higher tendency to form hydrogen bonds with PEG molecules. Chiboswi et al. observed variable adsorption behavior of PEGs on TiO_2_ surfaces with different affinities of different polymer chain lengths [23]; however, the binding sites may still be the same, but the total amount of adsorbed polymer will be higher for the higher-molecular-weight molecule. Moreover, changes in the conformation of the polymer as their surface concentration increases may lead to increased blockage and hydraulic resistance. 

### 3.3. Rejection Analysis 

**Figure 5:**
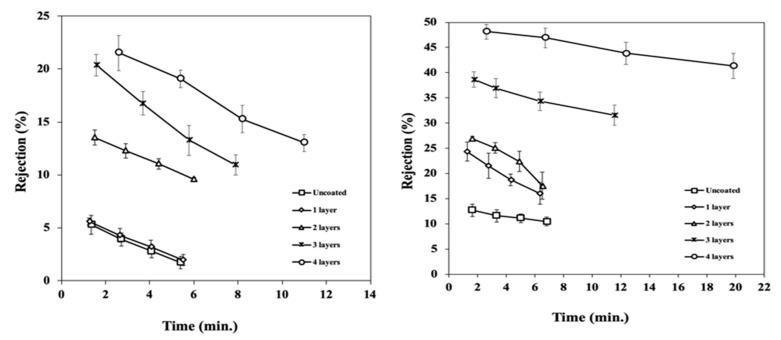
Rejection evolution vs. time: (**left**) 25 ppm solution PEG (MW 20,000 Da); (**right**) 25 ppm solution PEG (MW 200,000 Da); Q_feed_ = 33 mL/min; Q_retent =_ 22.5 mL/L; T = 25 °C.

The rejection of the coated membranes was investigated from the feed and permeate concentrations in the filtrations of the PEG feed solutions. The results are presented in Figure 5.

**Figure 6:**
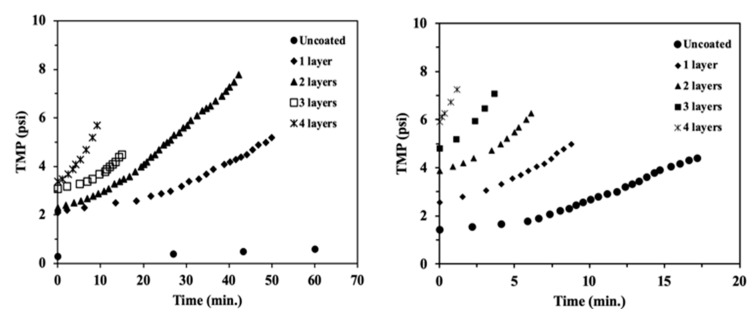
Transmembrane pressure vs. time during filtration of (**left**) 25 ppm PEG solution (MW 20 kDa); (**right**) 25 ppm PEG solution (MW 200 kDa); Q_feed_ = 33 mL/min; Q_retent_ = 22.5 mL/L; T = 25 °C.

A decrease in the apparent rejection (increase in the concentration of the permeate) over the first few minutes of the filtration was observed in most cases, in particular in the lower-MW PEG solution, without a stable condition measurement. At the same time, transmembrane pressure increased significantly, as shown by the steep rise in the curves shown in Figure 6, and it can be taken as an indication of the accumulation and potential adsorption of PEG molecules on the surface, as well as the onset of fouling and concentration polarization.

The first coating layer produced negligible to minimal changes in the retention capabilities of the support. Two to three layers were required to reject the test molecules, but only up to a modest percentage. Even for the four-layer coated membrane, rejection was 15% and 45% for the 20 kDa and 200 kDa molecules, respectively, which further indicates that macropores from the support were still exposed. The transmembrane pressure rapidly increased during these experiments, and the rate of increase was proportional to the number of layers, suggesting the formation of a fouling layer due to the accumulation of PEG molecules on the surface.

The blocking of the pores by the consecutive TiO_2_ condensed layers and the accumulation of the PEG on the surface of the membrane both contributed to the rejection results. The accumulated foulant cake, while increasing hydraulic resistance, has the potential to improve rejection. However, this was not observed in our experiments, where the accumulation of PEG may have blocked the smaller pores but not the larger pores in sections of the support material not completely covered by the CLD nanocoating; this allowed for the passage of the molecules near the surface and led to poor separation characteristics.

However, it is important to note that concentration polarization and fouling of the membranes are considered interferences in the determination of the actual rejection characteristics of the membrane. For macromolecules such as PEG, these effects may lead to higher apparent rejection due to filtration and attachment in the fouling layer, but they can also result in poor permeate quality due to a transmembrane pressure buildup while counteracting a severe decline in permeate flux. Therefore, the outcome of these experiments should not be regarded as indicative of the rejection characteristics of the filters, i.e., molecular weight cut off, but they are nevertheless informative as a probing tool to investigate the new materials.

## 4. Conclusions

Tubular alumina microfiltration ceramic membranes were subjected to surface coating by the condensed layer deposition of TiO_2_ nanoparticles. Multilayers were obtained by successive treatments of TiO_2_/PANI deposition.

Given the nanoscale of the particles deposited, the layer thicknesses were not evident from SEM; however, computer-assisted image analysis revealed a decrease in the range of sizes detected after each additional coating layer. Clean water flux measurements showed a similar trend, as hydraulic resistance increased as nanoparticles deposited on the surface. Therefore, these results confirmed that the coating process was successfully achieved in this work.

Filtration of the uncharged model molecules showed that rejection increased with the number of coatings but was incomplete for all tested materials. However, transmembrane pressure increased significantly during filtration and with the number of layers in the material, and thus additional coating cycles do not represent an attractive strategy to improve rejection. All coated membranes showed good permeability recoveries after hydraulic cleaning, most likely due to the weak surface interactions with Ti_2_O and more superficial fouling than for the bare supports.

These results could be interpreted to suggest that the TiO_2_ nanoparticles did not completely cover the pores on the membrane’s surface or fill the permeable paths in the support layer. This could be attributed to the roughness and relatively large surface pores of the support material; the inclusion of intermediate sections, for example by sol–gel processing, between the large-pore-size support material and the nanoscale CLD coating will most likely improve the integrity of the TiO_2_ layer, while adding complexity, cost, and an increase in the thickness that is detrimental to the overall permeability of the filter. 

The CLD technique can additionally find application in the fabrication of catalytic coatings in the emerging field of reactive membranes. The process allows for the creation of nanoscale layers that will minimize the non-surface-active mass of the catalyst, increasing efficiency and as well as uniformly distributing the catalyst over the support. The ability to create uniform, extra-thin active coating layers in asymmetric membrane minimizes the overall hydraulic resistance, but nanometer thicknesses are difficult or impractical to obtain with current methods. Although the durability of the materials was not systematically investigated, the results from repeated experimental runs, different tests, and routine handling of the membranes in the laboratory were remarkably reproducible, as can be seen, for example, by the error bars in Figure 5, suggesting the good adhesion and mechanical stability of the coatings. The coating process is conducted at room temperature in open an atmosphere with minimal waste of reactants; thus it compares positively with current coating techniques regarding cost, operation safety, environmental impact, and potential for the large-scale manufacturing of water treatment materials. 

## Figures and Tables

**Table 1:** Percentage permeability after coating of supports and filtration: P_sup_ = support material; P_mem_ = as-prepared coated membranes; P_feed1_ = after filtration of 20 kDa PEG solution and hydraulic cleaning; P_feed2_ = after filtration of 200 kDa PEG solution and hydraulic cleaning.

	Uncoated	1-Layer	2-Layer	3-Layer	4-Layer
P_mem_:P_sup_	-	73%	61%	49%	44%
P_feed1_:P_mem_	86%	90%	91%	92%	96%
P_feed2_:P_mem_	73%	79%	81%	85%	90%

## Data Availability

The data that support the findings of this study are available on request from the corresponding author, M.M.F.
